# Genetic analysis of elevated levels of creatinine and cystatin C biomarkers reveals novel genetic loci associated with kidney function

**DOI:** 10.1093/hmg/ddaf018

**Published:** 2025-02-10

**Authors:** Matteo D’Antonio, Timothy D Arthur, Wilfredo G Gonzalez Rivera, Ximei Wu, Jennifer P Nguyen, Melissa Gymrek, Park Woo-Yeong, Kelly A Frazer

**Affiliations:** Division of Biomedical Informatics, Department of Medicine, University of California San Diego, 9500 Gilman Dr., La Jolla, CA 92093, United States; Division of Biomedical Informatics, Department of Medicine, University of California San Diego, 9500 Gilman Dr., La Jolla, CA 92093, United States; Biomedical Sciences Graduate Program, University of California, San Diego, 9500 Gilman Dr., La Jolla, CA 92093, United States; Division of Biomedical Informatics, Department of Medicine, University of California San Diego, 9500 Gilman Dr., La Jolla, CA 92093, United States; Bioinformatics and Systems Biology Graduate Program, University of California, San Diego, 9500 Gilman Dr., La Jolla, CA 92093, United States; Division of Biomedical Informatics, Department of Medicine, University of California San Diego, 9500 Gilman Dr., La Jolla, CA 92093, United States; Division of Biomedical Informatics, Department of Medicine, University of California San Diego, 9500 Gilman Dr., La Jolla, CA 92093, United States; Bioinformatics and Systems Biology Graduate Program, University of California, San Diego, 9500 Gilman Dr., La Jolla, CA 92093, United States; Division of Biomedical Informatics, Department of Medicine, University of California San Diego, 9500 Gilman Dr., La Jolla, CA 92093, United States; Department of Computer Science and Engineering, University of California San Diego, 9500 Gilman Dr., La Jolla, CA 92093, United States; Department of Medicine, University of California San Diego, 9500 Gilman Dr., La Jolla, CA 92093, United States; Division of Nephrology, Department of Internal Medicine, Keimyung University School of Medicine, Keimyung University Dongsan Hospital, 1035 Dalgubeol-daero, Daegu, Republic of Korea; Department of Pediatrics, University of California San Diego, 9500 Gilman Dr., La Jolla, CA, 92093, United States; Institute of Genomic Medicine, University of California San Diego, 9500 Gilman Dr, 9500 Gilman Dr., La Jolla, CA 92093, United States

**Keywords:** genome-wide association studies, creatinine, cystatin C, kidney function, chronic kidney disease

## Abstract

The rising prevalence of chronic kidney disease (CKD), affecting an estimated 37 million adults in the United States, presents a significant global health challenge. CKD is typically assessed using estimated Glomerular Filtration Rate (eGFR), which incorporates serum levels of biomarkers such as creatinine and cystatin C. However, these biomarkers do not directly measure kidney function; their elevation in CKD results from diminished glomerular filtration. Genome-wide association studies (GWAS) based on eGFR formulas using creatinine (eGFRcre) or cystatin C (eGFRcys) have identified distinct non-overlapping loci, raising questions about whether these loci govern kidney function or biomarker metabolism. In this study, we show that GWAS on creatinine and cystatin C levels in healthy individuals reveal both nonoverlapping genetic loci impacting their metabolism as well as overlapping genetic loci associated with kidney function; whereas GWAS on elevated levels of these biomarkers uncover novel loci primarily associated with kidney function in CKD patients.

## Introduction

The prevalence of chronic kidney disease (CKD) is rising worldwide, driven by increasing rates of chronic conditions such as diabetes and hypertension, as well as an aging population. In the United States alone, CKD is estimated to affect 37 million adults [1, 2]. CKD is typically assessed and staged based on estimated Glomerular Filtration Rate (eGFR) values, which measure the kidney’s efficiency in filtering waste from the blood. Serum creatinine and/or cystatin C levels, combined with factors such as race, age, and sex, are commonly used to calculate eGFR and evaluate kidney function [3–5]. However, neither creatinine nor cystatin C are directly associated with kidney function, rather they serve as biomarkers whose levels are elevated in CKD due to reduced glomerular filtration. The lack of specific kidney function and CKD biomarkers has limited the development of drugs for CKD [6].

Genome-wide association studies (GWAS) involving over one million individuals have identified hundreds of genetic loci associated with eGFR [7–10]. Studies have shown that using different eGFR formulas based on creatinine (eGFRcre) or cystatin C (eGFRcys) can result in the discovery of distinct genetic loci [8, 9]. For example, the *CST3* locus, which encodes cystatin C, is identified only in eGFRcys GWAS, while the *GATM* locus, associated with creatinine levels and CKD [9, 11–13], is identified only in eGFRcre GWAS [10]. This discrepancy aligns with the distinct biological roles of these biomarkers. Creatinine is a waste product from the normal breakdown of creatinine phosphate in muscles, produced at a constant rate and filtered by the kidneys [14]. While creatinine levels are primarily associated with muscle mass and metabolism, they are also linked to lung function, specifically forced vital capacity (FVC) and forced expiratory volume in one second (FEV1) [15–17]. Conversely, cystatin C regulates the activity of cysteine proteases to prevent excessive protein degradation and maintain cellular homeostasis [18]. By controlling protease activity, cystatin C influences processes such as tissue remodeling, wound healing, and extracellular matrix turnover [18]. Cystatin C levels are associated with various cardiovascular risk factors, including dyslipidemia, cholesterol levels, obesity, and metabolic syndrome [19]. Given these differences, it is unclear what fraction of the genetic loci identified in eGFR GWAS are involved in kidney function and the development of CKD, or merely reflect the metabolism of the biomarkers used to measure eGFR.

Both the eGFRcre and eGFRcys formulas have been used in the clinic, and studies have shown that each has unique strengths and limitations in estimating kidney function across different populations [5, 20, 21]. For instance, the eGFRcys formula provides a more accurate estimate in individuals with lower levels of kidney function, offering a critical advantage in clinical contexts where precise monitoring of declining kidney function is necessary [20]. Conversely, other studies have highlighted discrepancies in eGFRcys estimates when applied to younger and healthier individuals, where traditional creatinine-based formulas (eGFRcre) may yield different results [5]. These recognized biases have driven the development of a new, combined formula (eGFRcrecys) that incorporates both creatinine and cystatin C levels [22]. This integrated approach aims to balance the strengths of each biomarker, providing a more reliable and comprehensive assessment of kidney function across diverse clinical scenarios and population groups.

We set out to determine if GWAS on elevated serum levels of creatinine and cystatin C would reveal genetic loci involved in kidney function and disease that were distinct from loci identified by GWAS on physiologically normal levels of these biomarkers. We conducted 60 GWAS for six traits (four eGFR formulas, creatinine levels and cystatin C levels) in individuals stratified by eight different levels of the creatinine biomarker or CDK status (disease, healthy). We show that GWAS conducted on individuals in the top1 percentile and bottom99 percentile identify distinct sets of genetic loci involved in kidney function, which are respectively strongly correlated with genetic loci found in GWAS conducted independently in either CKD patients or healthy individuals.

## Results

### eGFRcrecys values negatively correlate with biomarker levels

To examine variation in creatinine levels, cystatin C levels, and eGFR values, we analyzed measurements from 421 832 individuals of European ancestry in the UK Biobank (UKBB). Given the development of multiple eGFR formulas over the past 15 years, we first examined correlations between eGFRcre (2009), eGFRcre (2021), eGFRcys, and eGFRcrecys values (Fig. S1). The highest correlation was between the two eGFRcre formulas (R = 0.998) and the lowest between the eGFRcre and eGFRcys formulas (R = 0.634 and R = 0.628). The observed differences between eGFRcre and the eGFRcys values likely stem from uncorrelated variations in creatinine and cystatin C levels among healthy UKBB participants, which is consistent with previous studies [5, 22]. eGFRcrecys was highly correlated with the three other formulas (R = 0.834–0.931), reflecting its integration of both creatinine and cystatin C in estimating glomerular filtration rate, and hence general utility [22].

**Figure 1:**
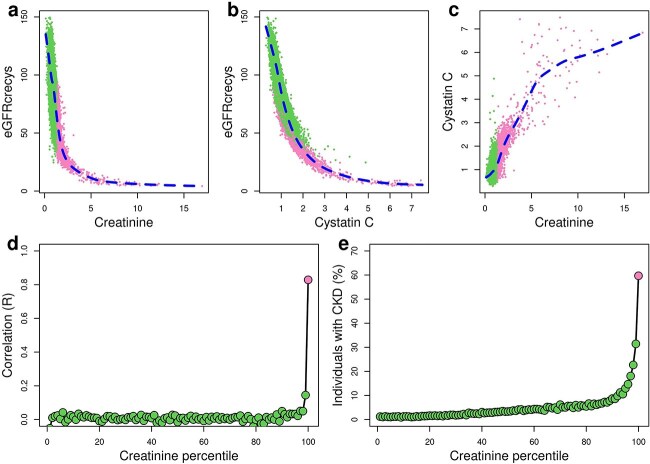
Associations between creatinine levels, cystatin C levels, eGFRcrecys (2021) and CKD, a-c) scatterplots showing the correlations between a) creatinine (mg/dl) and eGFRcrecys; b) cystatin C (mg/dL) and eGFRcrecys; and c) creatinine (mg/dL) and cystatin C (mg/L) levels. Each dot represents the indicated values for a UKBB individual. Individuals in the bottom99 (green) and top1 (pink) percentiles of creatine levels are indicated. Blue lines represent interpolation lines generated using the *smooth.Spline* function in R. d-e) Plots showing at each percentile of creatinine levels d) the correlation between creatinine and cystatin C levels; and e) the percentage of individuals with CKD. Individuals in the bottom99 (green) and top1 (pink) percentiles of creatine levels are indicated.

We assessed the correlations between eGFRcrecys values, creatinine levels, and cystatin C levels. Both biomarkers showed a strong negative correlation with eGFRcrecys (creatinine: r = −0.570; and cystatin C: r = −0.850, Fig. 1a and b), which results from the formula including the product of serum creatinine and cystatin C levels, both with negative exponents [22]. Hence, the eGFRcrecys values flatten out at extremely high creatinine and Cystatin C levels (Fig. 1a and b).

### Elevated levels of creatinine and cystatin C biomarkers correlated with each other and CKD

We next investigated the correlations between creatinine levels, cystatin C levels and CKD status in the 421 832 UKBB individuals. Creatinine and cystatin C levels demonstrated a positive significant correlation (r = 0.637, Fig. 1c). However, the correlation appeared to be primarily driven by individuals with high levels of both biomarkers. To investigate this further, we ranked individuals based on their creatinine levels because this measurement is routinely used to assess kidney function and hence widely available, and then examined how the correlation varied across different creatine percentiles. We observed no correlation in the bottom 98 percentiles (median = 0.0105, Fig. 1d), moderate correlation in the 99^th^ percentile (r = 0.145), and strong correlation in the 100^th^ percentile (r = 0.829). The correlation between creatinine and cystatin C levels at each percentile were strongly correlated with the fraction of UKBB individuals diagnosed with CKD (r = 0.847, Fig. 1e). As expected, the individuals with the highest creatinine level percentiles tend to be CKD patients: in the 100^th^ percentile, 59.7% individuals were diagnosed with CKD, 31.4% in the 99^th^ percentile and 22.7% in the 98^th^ percentile, and only the top eight percentiles had at least 10% individuals diagnosed with CKD (Table S1). Overall, individuals in the top 100th percentile (named ‘top1’ dataset hereafter, 59.7%) for creatinine levels were 13.4 times more likely to be diagnosed with CKD than individuals in the bottom 99 percentiles (bottom99, 4.48%, Table 1), indicating that, as expected, high creatinine levels correspond to increased risk for CKD. These observations also suggest that the strong correlation between creatinine and cystatin C levels only in the top1 dataset is due to both these metabolites not being correctly filtered by kidneys with impaired function.

**Table 1:** Population characteristics.

**data**	**bottom99**	**top1**
Sample size^a^	417 815	4017
Age^b^	56.7 +/− 8.02	61.4 +/− 6.96
Male (%)^a^	190 043 (45.5%)	3382 (84.2%)
Creatinine (mg/dL)^b^	0.809 +/− 0.154	1.71 +/− 0.977
Cystatin C (mg/L)^b^	0.902 +/− 0.144	1.58 +/− 0.744
eGFRcrecys^b^	95.2 +/− 13.8	50.3 +/− 16.2
Chronic kidney disease^a^	18 725 (4.48%)	2408 (59.9%)
Type 2 diabetes^a^	3718 (0.89%)	284 (7.07%)
Hypertension^a^	126 865 (30.4%)	2907 (72.4%)
Acute myocardial infarction^a^	18 326 (4.39%)	725 (18%)
Heart failure^a^	13 164 (3.15%)	803 (20%)
Atherosclerosis^a^	2705 (0.647%)	229 (5.7%)

^a^Number of individuals in the bottom 99 and top 1 percentiles of creatine levels

^b^The mean and standard deviation are reported

### Creatinine levels are markers for other chronic diseases

**Figure 2:**
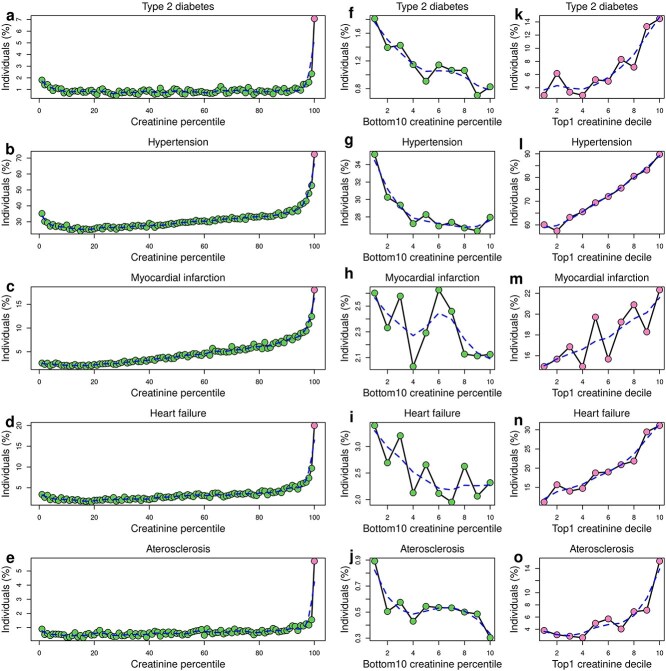
Associations between creatinine levels and other chronic diseases, plots showing the correlations between serum creatinine levels and the risk for five chronic diseases: a-e) across all individuals; f-j) in individuals in the bottom decile for creatinine levels (bottom10); and k-o) in individuals in the top1 dataset. Dashed lines represent interpolation lines generated using the *smooth.Spline* function in R.

Since the major causes of chronic impaired kidney function and CKD are type 2 diabetes, hypertension and cardiovascular disease, we investigate whether high serum creatinine levels are also associated with higher risk for these diseases. Specifically, we tested the correlations of creatinine levels with type 2 diabetes, essential hypertension, acute myocardial infarction, heart failure and atherosclerosis. In general, we observed a similar trend as with CKD (Fig. 1e), with the top creatinine percentiles associated with increased disease risk, and the top1 dataset having the highest risk (Fig. 2a–e, Table 1). Hypertension having the smallest increase in the top1 dataset compared with the bottom99 (2.38 times: 30.4% in the bottom99 compared to 72.4% in the top1), and atherosclerosis had the largest (8.80 times: 0.65% in the bottom99 compared to 5.7% in the top1). Interestingly, we found that the bottom ten creatinine percentiles (bottom10) had the opposite trend, with a negative association between creatinine levels and disease risk (Fig. 2f–j, Table S2). This increase in chronic disease risk at extremely low creatinine levels may indicate individuals with poor general health, as low creatinine levels are a marker for low muscle mass and malnourishment [23].

**Figure 3:**
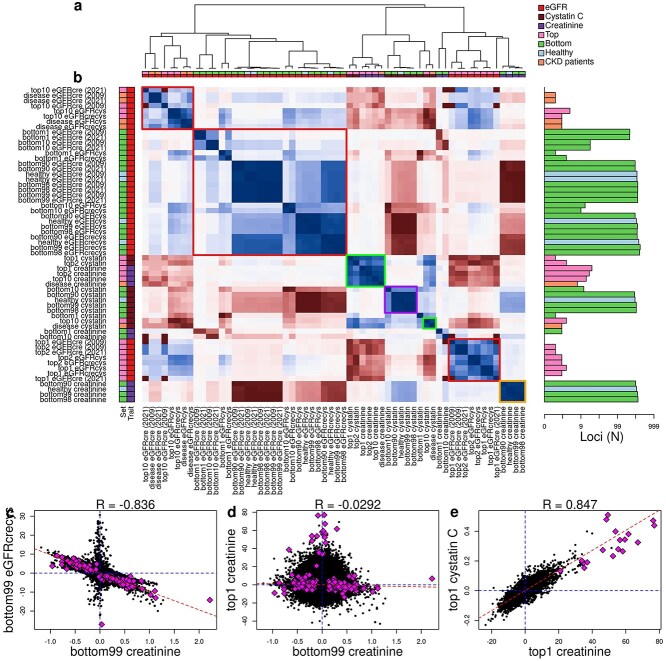
Correlations of GWAS effect sizes a-b) Dendrogram and heatmap showing the effect size correlations between each pair of GWAS. On the right, a barplot (X axis in log scale) showing the number of genome-wide significant loci is displayed. Colored squares represent six clusters (top left to bottom right): 1) eGFR GWAS for top10 set of individuals and CKD patients; 2) eGFR GWAS for individual in lower percentiles and healthy individuals; 3) top1, top2 and disease clusters for creatinine and cystatin C; and 3) bottom clusters for cystatin C; 4) top1 and top2 clusters for eGFR; and 5) bottom clusters for creatinine. Color scale represents correlation levels. Items were sorted using the *hclust* function in R to cluster studies that correlate with each other c-e) scatter plots showing the correlation between trait GWAS effect sizes for: c) creatinine versus eGFRcrecys in the bottom99 set of individuals; d) creatinine in top1 versus bottom99 set of individuals; and e) creatinine versus cystatin C in the top1 set of individuals. Diamonds represent all lead variants (*P* < 1 × 10^−8^).

We further stratified the 4017 individuals in the top1 dataset based on creatinine levels and investigated if the positive relationship between higher creatinine levels and increased risk for all five chronic diseases was observable in this set. We found a significant positive association for all diseases but acute myocardial infarction (*P* = 0.090, Fig. 2k–o, Table S2). These results suggest that, even with a relatively small sample size (4017 individuals) that includes individuals with extremely high creatinine levels, we can stratify chronic disease risk.

In summary, elevated creatinine levels result from reduced glomerular filtration, making them a key biomarker for chronic kidney disease (CKD). They are also associated with type 2 diabetes, hypertension, and cardiovascular disease. However, it remains unclear whether these associations arise in part because these chronic disease conditions themselves raise creatinine levels, or if the observed correlations are solely due to the fact that they are risk factors for CKD, and kidney impairment results in increased levels of the biomarker. If the former (i.e. independently raise creatinine levels), GWAS on the top1 dataset could identify genetic loci associated with the risk of developing impaired kidney function and CKD in individuals with chronic metabolic diseases. In the following section, for simplicity we assume that the identified genetic loci are associated with kidney function and disease, but discuss the confounding effects of risk factors such as type 2 diabetes and hypertension in later sections.

### GWAS loci in top1 and bottom99 datasets respectively correlate with CKD and healthy individuals

We sought to examine the correlation of genetic loci identified by GWAS conducted on a range of creatinine and cystatin C levels as well as CKD status (disease, healthy). Given the fact that creatinine levels and cystatin C are only correlated at elevated levels (Fig. 1c and d), we hypothesized that conducting GWAS on individuals with physiologically normal levels would identify a combination of non-overlapping genetic loci associated with the metabolism of these biomarkers. And that GWAS performed on individuals with extremely elevated levels of creatinine and cystatin C would identify genetic loci primarily associated with the risk of developing impaired kidney function and CKD. Further, the negative correlation of eGFRcrecys values compared with creatinine levels and cystatin C levels (Fig. 1a and b), suggest that GWAS for these traits should identify genetic loci with opposite effect sizes.

To test these hypotheses, we performed a total of 60 GWAS using the 421 832 UKBB individuals (Fig. 3). We conducted GWAS on six traits: 1) creatinine levels, 2) cystatin C levels, 3) eGFRcre (2009) values, 4) eGFRcre (2021) values, 5) eGFRcys values, and 6) eGFRcrecys values; which were stratified based on eight creatinine levels (individuals in the top 1, 2 and 10 percentiles, and the bottom 1, 10, 90, 98 and 99 percentiles) as well as CKD status (disease, healthy). Overall, we identified 836 independent loci (Table S3). The GWAS with the most genome-wide significant loci was for eGFRcrecys in individuals in the bottom 99 percentile of creatinine levels (bottom99, 419 loci, Fig. 3a and b), followed by eGFRcrecys in the bottom 98 percentiles (bottom98, 406 loci), and eGFRcrecys in healthy individuals (384 loci). Conversely, the GWAS with the smallest number of loci were for eGFR formulas in individuals in the top 1 percentile (top1) and top 2 percentile (top2), all with only three or fewer loci. This is to be expected, considering that all eGFR formulas flatten eGFR distributions towards zero at high creatinine or cystatin C levels (Fig. 1a and b). Overall, we observed a low overlap between loci across the 60 GWAS, with the *CST3* locus (chr20:20933145–26 316 464), which encodes for cystatin C, being the one shared across the most studies (31 GWAS), followed by chr13:48391356–51 421 153 (29 GWAS), which has previously been associated with both creatinine and cystatin C levels [24, 25], and the major histocompatibility complex (MHC, 28 GWAS). These results are in part due to different statistical power across the different GWAS (Fig. S2), as their sample sizes span two orders of magnitude (top1 set: 4017 individuals; bottom99 set: 417815).

To investigate the overlap between loci across the 60 GWAS we calculated pairwise correlation of effect sizes rather than counting shared genome-wide significant loci because of their different statistical powers (Fig. 3a and b, Table S4). GWAS loci effect sizes were in general positively correlated across all eGFR formula GWAS (red squares in Fig. 3b) and negatively correlated with GWAS loci on creatinine and cystatin C levels. Specifically, we observed three distinct eGFR GWAS clusters, which displayed weak positive correlation with each other. One cluster contained GWAS for all eGFR formulas on individuals in the top 10 percentile of creatinine levels (top10) and CKD patients (disease). The second cluster contained GWAS for all eGFR formulas on individuals with lower creatinine levels (bottom 1, 10, 90, 98 and 99 percentiles) and healthy individuals (without CKD). While the third cluster contained GWAS for all GFR formulas on individuals in the top1 and top2 percentiles. This analysis shows that GWAS for all eGFR formulas across individuals with varying creatinine levels and CDK status identify a set of overlapping loci that have correlated effect sizes; however, the loci effect sizes are most correlated between GWAS conducted on individuals in the top10 creatinine level percentile and CKD patients as well as between GWAS conducted on individuals in the bottom creatinine level percentiles and healthy individuals.

Examining the pairwise correlation of GWAS loci effect sizes for creatinine and cystatin C levels resulted in four clusters Fig. 3a and b). Overall, the GWAS for creatinine levels in individuals in the bottom percentiles and healthy individuals clustered together (orange square in Fig. 3b). Likewise, the GWAS for cystatin C levels in individuals in the bottom percentiles and healthy individuals clustered together (purple square in Fig. 3b). Although these clusters were distinct indicating that they were composed of GWAS loci associated with either creatinine metabolism or cystatin C metabolism, they were correlated (Fig. 3b) indicating that some of the GWAS loci were likely associated with kidney function. GWAS loci for creatinine and cystatin C levels conducted on individuals in the top percentiles and CKD patients formed two clusters that were correlated with each other (green squares in Fig. 3b). Overall, the effect sizes of GWAS loci for the biomarkers were negatively correlated with the GWAS loci for eGFR (Fig. 3b and c), and the effect sizes of GWAS loci for the top and bottom percentiles of the two biomarker traits were not correlated (Fig. 3d). Of note, while the GWAS loci for the three different traits (eGFR, creatinine and cystatin C) tended to cluster independently, the loci identified for creatinine and cystatin C levels in individuals in the top percentiles (top1 and top2) were strongly correlated and clustered with loci for the creatinine GWAS in CKD patients (Fig. 3b and e).

In summary, our findings suggest that GWAS for eGFR, creatinine and cystatin C in individuals with low creatinine levels (we will use the bottom99 set hereafter) identifies loci respectively associated with normal kidney function, creatinine metabolism and cystatin C metabolism. Whereas GWAS for creatinine and cystatin C levels in individuals with high serum creatinine (we will use the top1 set hereafter) identify loci associated with impaired kidney function because the serum levels of these two metabolites are both increased as a consequence of kidney disease. Finally, because eGFR values are flattened at extremely high creatinine levels and hence variability is low (Fig. 1b and c), the GWAS on the eGFR formulas in individuals with high creatinine levels does not identify many genome-wide significant loci (Fig. 3b).

### Bottom99 GWAS includes both biomarker metabolism and kidney function genetic associations

**Figure 4:**
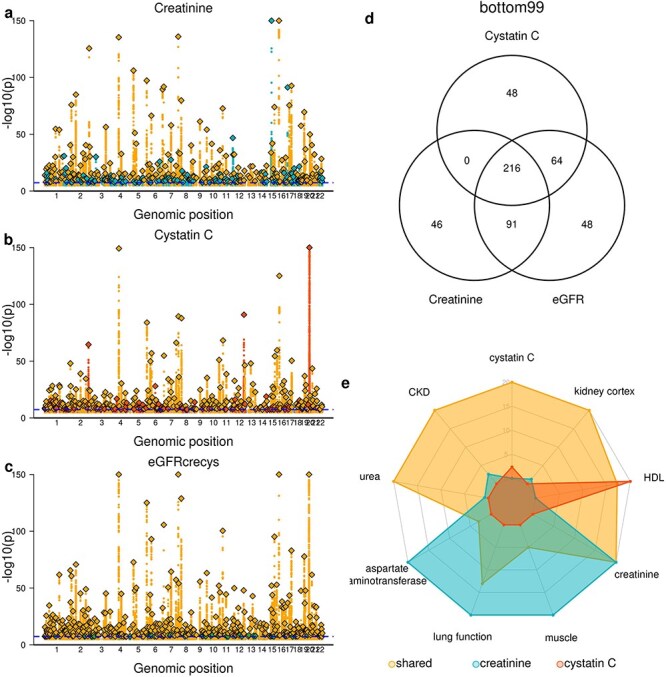
GWAS of creatinine, cystatin C, and eGFRcrecys traits in the bottom99 set of individuals a-c) Manhattan plots showing the associations between genetic variation and a) creatinine, b) cystatin C, and c) eGFRcrecys measurements in the bottom99 set of individuals. Loci in orange are shared across all three traits, loci in blue are genome-wide significant for creatinine but not cystatin C (panel a), and loci in red are genome-wide significant for cystatin C but not creatinine (panel b). d) Venn diagram showing the overlap between creatinine, cystatin C, and eGFRcrecys GWAS loci in the bottom99 set of individuals. e) Radar plot showing functional enrichment for GWAS loci specific for creatinine, cystatin C, or shared. Axis represents -log_10_ (p-value). Full functional enrichment analysis results are shown in Table S4.

We further examined the GWAS loci for the eGFRcrecys, creatinine and cystatin C traits in the bottom99 set of individuals to gain deeper insight into their biological functions (Table S1). Overall, we detected 513 loci (*P* < 5x10^−8^), including 216 (42.1%) shared (LD R^2^ > = 0.8) across all three traits (creatinine, cystatin C, and eGFRcrecys), 155 shared between two (30.2%), and the remaining 142 (27.7%) specific for a single trait (Fig. 4a and d, Table S3). Since eGFRcrecys was calculated using both creatinine and cystatin C levels, we observed no loci shared between creatinine and cystatin C but not eGFRcrecys. Most of the creatinine loci (265 of 368, 72.0%) either had been associated with creatinine levels in the GWAS catalog [26], were in strong LD with variants associated with serum creatinine levels in the GWAS catalog, or overlapped genes that have been described as associated with serum creatinine levels in the GWAS catalog (Table S5). Two of the strongest creatinine loci overlapped with eGFRcrecys loci but not with cystatin C loci, including the *GATM* locus (lead variant rs76825670) on chromosome 15, associated with creatinine levels and CKD [9, 11–13], and the *SLC47A1* locus (lead variant rs111653425) on chromosome 17, which has also been associated with both creatinine levels and eGFR, but not cystatin C [8, 24]. Of the 336 cystatin C loci, 48 (14.3%) were not shared with creatinine or eGFRcrecys and only 40.1% (135 of 336) had been previously described in cystatin C GWAS (Table S5). Two of the three strongest cystatin C signals were shared with eGFRcrecys but not with creatinine, including the *CST3* locus (lead variant rs734801) on chromosome 20, which encodes for cystatin C, and a locus on chr12q24.11 between *SH2B3* and *ATXN2* (lead variant rs3184504), that have been previously associated with cystatin C and many other traits, including blood pressure, cholesterol and hemoglobin levels, but not creatinine or kidney function [27–29].

We characterized the loci for GWAS conducted on the bottom99 set of individuals by conducting a comprehensive functional enrichment analysis on the genes independently associated with loci for creatinine and cystatin C, as well as ‘shared’ genes associated with overlapping loci for cystatin C and either creatinine and/or eGFRcrecys (Table S6). We annotated each locus with the gene closest to its lead variant, then performed functional enrichment analysis on gene sets associated with gene expression in relevant tissues and cell types, gene function, pathways and genetic associations against the rest of human genes using *enrichr* [30]. Our findings revealed novel insights into the distinct biological roles of these loci. As expected, the loci associated with creatinine but not with cystatin C demonstrated enrichment for traits related to lung function, as well as aspartate aminotransferase GWAS, and genes overexpressed in muscle [31] (Fig. 4e, Table S6). This aligns with the established role of creatinine as a byproduct of creatinine metabolism, primarily found in striated muscle, where it serves as an energy reserve [19, 32]. Similarly, the loci specific to cystatin C were associated with GWAS traits such as high-density lipoprotein (HDL) cholesterol levels, consistent with cystatin C's known associations with dyslipidemia and diabetic nephropathy in patients with type 2 diabetes [19]. In contrast, the GWAS loci shared between cystatin C and creatinine and/or eGFRcrecys exhibited robust enrichment not only for both biomarkers, but also for signals associated with kidney function, including CKD and blood urea levels [33]. Furthermore, these shared loci demonstrated a significant enrichment for genes expressed in the kidney cortex, further supporting their association with renal physiology [34].

Together, these observations underscore the multifaceted nature of biomarker metabolism and kidney function regulation; while creatinine-specific and cystatin C-specific loci play distinct roles in biomarker metabolism, many of the loci shared between creatinine and cystatin C play roles in kidney function, gene expression and disease. While previous studies have shown cystatin C levels associated with chronic diseases such as type 2 diabetes and hypertension [19], it is not known whether these associations are due to impaired kidney function caused by these diseases or whether there is a direct link between cystatin C levels and other biomarkers such as cholesterol. Our findings that cystatin C-specific loci are associated with HDL levels in the bottom99 dataset supports previous observations [35] suggesting that these associations represent a direct link.

### GWAS in the top1 dataset identify novel kidney disease loci

**Figure 5:**
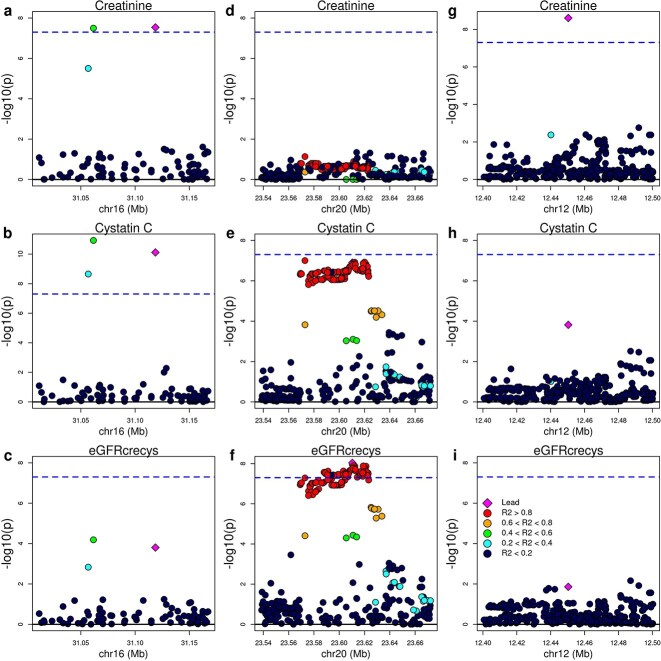
GWAS loci in the top1 dataset. Manhattan plots showing for three loci (a-c, d-f, g-i) the associations between genetic variation and three GWAS traits (creatinine levels, cystatin C levels, and eGFRcrecys) in the top1 set of individuals. The loci represented in set of panels include: a-c) *BCKDK*, d-f) *CTS3*, and g-i) *RNU6-1295P*. The lead variants are shown as diamonds.

We set out to examine if the GWAS loci for the eGFRcrecys, creatinine and cystatin C traits in the top1 set of individuals are associated with impaired kidney function. In total, across the three traits we identified 22 GWAS variants (*P* < 5 × 10^−8^), of which 18 were creatinine-specific (Table 2), two (rs117766531 for creatinine and rs116872173 for cystatin C) were in the same locus on chromosome 16 (Fig. 5a–c), and two were eGFRcrecys-specific (Fig. S3, Table S5). Most of the GWAS variants had relatively low minor allele frequencies (1% < MAF < 5%) and high effect sizes (Table 2). Given that the statistical power in the top1 datasets was relatively low due to the small sample size (4017 individuals), we investigated the p-values of the two eGFRcrecys-specific signals, and found that one (rs9532933 on chromosome 13) was borderline significant for the other two traits (*P* = 1.1 × 10^−6^ for creatinine and 3.0 × 10^−7^ for cystatin C, Table S7), while the other (rs116542696 on chromosome 20) was borderline significant only for cystatin C (*P* = 0.20 for creatinine and 1.3 × 10^−7^ for cystatin C, Fig. 5d–f, Table S7), suggesting that these signals are likely not eGFRcrecys-specific, but shared with at least one for the two biomarkers. We manually inspected the 21 GWAS loci and found that the lead variants were all novel as none had not been described in the GWAS catalog for eGFR, CKD, creatinine or cystatin C levels; but two loci specific for creatinine (lead variants: rs139912558 on chromosome 11 overlapping *AC090791.1*, and rs117489454 on chromosome 12 overlapping *RNU6-1295P*) and one locus for eGFRcrecys (rs116542696 overlapping *CST3*) overlapped with the 513 GWAS loci identified in the bottom99 dataset (Table 2).

**Table 2:** Lead variants in the top1 datasets.

**Trait**	**Chromosome**	**Position**	**Reference allele**	**Alternative allele**	**rsID**	**MAF (%)**	**Overlapping gene**	**Effect size**	**Standard error of beta**	**-LOG10(p)**
creatinine	chr1	90 554 838	G	A	rs142700093	1.15	*AL161797.1*	53.6	9.6	7.6
creatinine	chr11	29 193 997	A	G	rs139912558	2.60	*AC090791.1*	34.4	6.1	7.8
creatinine	chr12	12 450 399	T	G	rs117489454	2.65	*RNU6-1295P*	34.8	5.8	8.6
creatinine	chr12	66 998 356	CT	C	12:66998356_CT_C	2.29	*GRIP1*	55.5	9.3	8.6
eGFRcrecys	chr13	42 411 444	C	T	rs9532933	1.92	*VWA8*	−54.3	9.7	7.7
creatinine	chr14	77 954 019	C	T	rs79410779	3.04	*ISM2*	33.8	5.7	8.3
creatinine	chr15	69 850 894	G	T	rs192277306	1.08	*DRAIC*	61.6	10.1	9.0
creatinine	chr15	91 899 463	G	A	rs111703902	1.32	*AC123784.1*	53.9	9.7	7.6
cystatin	chr16	31 061 606	T	C	rs116872173	1.06	*AC135050.3*	0.5	0.1	10.9
creatinine	chr16	31 118 690	G	A	rs117766531	1.05	*BCKDK*	48.4	8.7	7.5
creatinine	chr18	29 589 641	C	T	rs9958131	1.56	*RNF125*	67.2	12.0	7.6
eGFRcrecys	chr20	23 610 381	A	T	rs116542696	22.10	*CST3*	18.2	3.2	8.0
creatinine	chr3	13 309 923	T	A	rs116309865	1.26	*IQSEC1*	58.2	9.8	8.4
creatinine	chr3	74 674 979	CACACACAA	C	3:74674979_CACACACAA_C	1.25	*AC128653.1*	59.5	10.5	7.8
creatinine	chr3	1.07E+08	G	A	rs138126844	1.05	*AC063944.1*	56.5	9.7	8.2
creatinine	chr4	75 577 874	G	T	rs143722587	2.19	*AC142293.3*	37.0	6.4	8.1
creatinine	chr5	1.75E+08	A	T	rs62390582	1.18	*ARL2BPP6*	52.4	9.3	7.7
creatinine	chr6	75 340 254	T	C	rs79292697	7.35	*AL357507.1*	20.9	3.7	7.7
creatinine	chr7	70 966 280	C	T	rs117032381	2.20	*GALNT17*	35.3	6.4	7.4
creatinine	chr7	1.58E+08	C	G	rs142100108	1.59	*PTPRN2*	46.3	8.0	8.2
creatinine	chr8	7 693 474	C	G	rs529832206	1.24	*DEFB104A*	77.0	13.4	8.0
creatinine	chr9	67 917 314	C	T	rs534922938	1.36	*BX649567.1*	76.4	12.8	8.6

To further investigate the 21 GWAS loci, we mapped the lead variant at each locus with its closest gene and investigated whether the gene had previously been described in the GWAS catalog as associated with serum levels of creatinine, cystatin C or eGFR, or if the lead variant was in strong LD (R^2^ > 0.8) with variants that had either been associated any of these three traits or overlapped genes that had been described as associated (Table S5). We noted that several genes at these loci have kidney-related functions or have been associated with chronic diseases that impact kidney function, including type 2 diabetes, obesity and atherosclerosis.

Of the 18 creatinine-specific loci (Table S2), multiple had kidney-associated functions: 1) *RNU6-1295P* (RNA, U6 small nuclear 1295, pseudogene) located 23 kb upstream of *LRP6*, which encodes for an LDL receptor-related protein, whose knockout in mice results in impaired renal development and cystic dysplasia [36], and has been associated with obesity and atherosclerosis [37] (Fig. 5g–i); 2) *IQSEC1*, a gene involved in epithelial to mesenchymal transition, and for which hypermethylation is associated with decreased kidney function and CKD [38]; 3) *PTPRN2* involved in type 2 diabetes and obesity and associated with diabetic renal disease [39]; 4) *AC128653.1* a non-coding RNA located in a *CNTN3* intron, a gene downregulated in kidney cancer, compared with normal tissues [40]; and 5) *GRIP1* involved in renal agenesis [41]. While the *GRIP1* locus has been previously associated through GWAS to creatinine levels in East Asians [24], the lead variant we identified (a 1-bp deletion at 12:66998356, rs5798830) is located 600 kb upstream of the GWAS lead variant (rs1658780), which is also located in an intron of *CAND1*. These two variants are not in LD (R^2^ < 0.01 and D′ = 0.41 in East Asians and 0.16 in Europeans), suggesting that they contribute to two distinct non-overlapping signals and potentially effect the expression of different genes. Finally, the interval encoding *GALNT17* has been associated with cognitive decline, educational attainment (notably lower in elderly populations), and warfarin use (commonly prescribed in older adults) [42], suggesting a role for this locus in the association between kidney function and other chronic diseases.

The locus shared between creatinine and cystatin C overlapped *BCKDK* (lead variant: rs117766531 on chromosome 16, Fig. 5a–c), a gene involved in acidosis and glucocorticoids metabolism, and catabolic signals associated with renal failure [43]. This locus has been previously associated through GWAS with Parkinson’s disease [44], response to warfarin treatment [45], and triglycerides levels [46], but not with kidney function or CKD.

A locus associated with eGFRcrecys overlapped the gene that encodes for cystatin C (*CST3*) on chromosome 20, Fig. 5d–f). This locus is associated with the strongest cystatin C signal in the bottom99 dataset and while cystatin C was not genome-wide significant in the top1 dataset, its p-value was borderline (1.3 × 10^−7^, Table S7) and was genome-wide significant in the top2 and top10 datasets (Table S3). However, the eGFRcrecys lead variant is not in the GWAS catalog and is not in LD with any cystatin C variants in the GWAS catalog, suggesting that this signal may be different than the cystatin C signal associated with *CST3* in the bottom99 dataset.

Altogether, these results show that stratifying individuals by their creatinine levels results in identifying novel loci associated with kidney function and suggest that conducting larger GWAS in the future on individuals with high creatinine levels would identify additional novel genes associated with kidney function and disease.

## Discussion

In this study, we initially observed that serum creatinine and cystatin C levels are only correlated in individuals with extremely high levels (Fig. 1a and d). We also noted that individuals with correlated serum creatinine and cystatin C levels were enriched for having CKD. Based on these observations we conducted 60 GWAS for six traits (four eGFR formulas, creatinine levels and cystatin C levels) in individuals stratified by eight different levels of the creatinine biomarker or CDK status (disease, healthy). The GWAS for all four eGFR formulas across the 10 different individual groups identified overlapping loci with correlated effect sizes; however, the loci effect sizes are most similar between GWAS conducted on individuals in the top creatinine level percentiles and CKD patients as well as between GWAS conducted on individuals in the bottom creatinine level percentiles and healthy individuals (Fig. 3a–b). The GWAS for creatinine and cystatin C levels conducted on individuals in the bottom percentiles and healthy individuals formed two distinct clusters suggesting that they are respectively composed of genetic loci associated with either creatinine metabolism or cystatin C metabolism. Whereas the GWAS for creatinine and cystatin C levels conducted on individuals in the top percentiles and CKD patients tended to cluster together. These observations suggested that GWAS for creatinine and cystatin C conducted in individuals with low percentiles identifies loci associated with metabolism of the biomarkers, where the GWAS for creatinine and cystatin C levels conducted in individuals with high percentiles predominantly identify loci associated with impaired kidney function.

We examined the GWAS loci of the creatinine, cystatin C and eGREcrecys traits conducted in individuals in the bottom99 percentiles in detail. The loci specific to either the creatinine or cystatin C GWAS in the bottom99 set exhibited distinct enrichments, reflecting their unique roles in biomarker metabolism and physiological functions; while the genetic loci shared between the two GWAS showed robust enrichment for kidney-related markers and diseases, highlighting their relevance to renal physiology.

On the other hand, the GWAS for creatinine or cystatin C in the top1 set proved instrumental in identifying novel genetic loci associated with kidney function. This dataset yielded unique genetic associations most of which were not observed in the bottom99 GWAS or previously identified in creatinine or CKD GWAS [7, 9, 10]. As expected, the top1 dataset was enriched for older individuals with a higher burden of chronic conditions, including cardiovascular diseases, diabetes, cancer, and obesity (Table 1), and some of the top1 loci for the serum creatinine trait overlapped with genes and regions previously implicated in age-related phenotypes, including: *GALNT17* with cognitive decline, educational attainment (notably lower in elderly populations), and warfarin use (commonly prescribed in older adults) [42]; *LRP6* with LDL levels, obesity and atherosclerosis [37]; and *PTPRN2* locus with type 2 diabetes and obesity [39]. These observations suggest that the genetic architecture underlying the serum creatinine trait in the top1 dataset may partially reflect pleiotropic effects related to aging and associated phenotypes. This overlap emphasizes the need to contextualize findings from this subgroup and consider the potential influence of survivor bias when interpreting these results. Future studies should aim to disentangle the effects of genetic variants on serum creatinine and other age-related conditions, ideally through longitudinal cohorts that can assess causality and temporal relationships.

In conclusion, our study shows that dividing biomarkers serving as a proxy for a disease state into physiologically normal and extreme levels can identify genetic loci that are associated with functions in the healthy and disease states of an organ. Specifically, we show that GWAS on the bottom99 set of individuals identified genetic loci active in kidney function of healthy individuals, while GWAS on the top1 set of individuals identified genetic loci active in the kidney function of CKD patients.

## Material and methods

### UKBB study cohort

For this study, we used 421 832 unrelated individuals of European genetic ancestry in the United Kingdom Biobank (UKBB). We limited the study to Europeans because average serum creatinine levels are strongly ancestry-related and GWAS of serum creatinine levels across multiple ancestries would be confounded. To obtain this cohort, we proceeded as follows:

We obtained data for 502 478 participants with imputed genotypes in the UKBB and removed related individuals using the genetic relatedness pairing information (UKBB Data Showcase Resource ID 22011). Specifically, each pairing of related participants is assigned a unique number and, for each pairing unique number, we retained only one individual. In total, we retained 479 806 unrelated individuals and to select Europeans, we annotated the ancestry of each individual based on their genotype principal component (PCA) coordinates (i.e. genetic ancestry) as follows:

1. We used the genotype PCA to annotate each individual with the most likely 1KGP superpopulation: 1) East Asian (EAS): Any other Asian background, Asian or Asian British, Chinese; 2) South Asian (SAS): Bangladeshi, Indian, Pakistani; 3) African (AFR): African, Any other Black background, Black or Black British, Caribbean; and European (EUR): Any other white background, British, Irish, White.
2. We calculated the centroid of each superpopulation in the genotype PCA space (top ten PCs) as the median value of all individuals that self-reported in each ethnicity group.
3. Next, we computed the distance of each individual from each superpopulation centroid. Each individual was annotated with its closest superpopulation centroid if the distance from the second-closest superpopulation was at least three times its distance from its closest superpopulation (Fig. S4). All individuals that did not satisfy this condition were labeled as ‘admixed’.
4. Of the 479 806, 2843 self-identified as ‘admixed’ (categories: Any other mixed background, Mixed, White and Asian, White and Black African, White and Black Caribbean) and 6627 as ‘unknown’. However, after annotating each individual with its closest super-population with the stringent filters described above, the number of admixed individuals increased to 18 931. The final study cohort was composed of 421 832 individuals with European genetic ancestry.

### Correlations between creatinine levels, cystatin C levels, eGFR, CKD and other chronic diseases

For each of the 421 832 participants, we obtained the following information: sex (data field 31), age at recruitment (21022), 40 genotype PCs (22009), self-reported ethnicity (21000), chronic renal failure as a measure of CKD (data field 132 032), type 2 diabetes (130706), essential hypertension (131286), acute myocardial infarction (131298), heart failure (131354), atherosclerosis (131380), creatinine (30700), and cystatin C (30720). Creatinine levels, reported in μmol/L, were converted to mg/dL.

We calculated the estimated glomerular filtration rate (eGFR) using four formulas [4, 5, 22, 47, 48]:

#### CKD-EPI formula, referred to as eGFRcre (2009) throughout manuscript, that uses only creatinine levels and has an ancestry component


\begin{align*} {eGFR}_{cr e(2009)}=141\times \mathit{\min}{\left(\frac{S_{cr}}{\kappa },1\right)}^{\alpha}\times \mathit{\max}{\left(\frac{S_{cr}}{\kappa },1\right)}^{-1.209}\\\times{0.993}^{age}\times 1.018\left[ if\ female\right]\times 1.159\left[ if\ AFR\right] \end{align*}

Where ${S}_{cr}$ is serum creatinine level in mg/dL; $\kappa$ is 0.7 for females and 0.9 for males; and $\alpha$ is −0.429 for females and − 0.411 for males. Given that we use only EUR individuals, we excluded the $\times 1.159$ associated with AFR individuals.

#### CKD-EPI formula, referred to as eGFRcre (2021) throughout manuscript, that uses only creatinine levels and has removed any ancestry component


\begin{align*} {eGFR}_{cr e(2021)}=142\times \mathit{\min}{\left(\frac{S_{cr}}{\kappa },1\right)}^{\alpha}\times \mathit{\max}{\left(\frac{S_{cr}}{\kappa },1\right)}^{-1.2}\\\times{0.9938}^{age}\times 1.012\left[ if\ female\right] \end{align*}

Where ${S}_{cr}$ is serum creatinine level in mg/dL; $\kappa$ is 0.7 for females and 0.9 for males; $\alpha$ is −0.241 for females and − 0.302 for males.

#### CKD-EPI formula, referred to as eGFRcys throughout manuscript, that uses only cystatin C levels


\begin{align*} {eGFR}_{cys}=133\times \mathit{\min}{\left(\frac{S_{cys}}{0.8},1\right)}^{-0.499}\times \mathit{\max}{\left(\frac{S_{cys}}{0.8},1\right)}^{-1.328}\\\times{0.996}^{age}\times 0.932\left[ if\ female\right] \end{align*}

Where ${S}_{cys}$ is serum cystatin C level in mg/L.

#### CKD-EPI formula, referred to as eGFRcrecys throughout manuscript, that incorporates both creatinine and cystatin C levels


\begin{align*} {eGFR}_{cr ecys}=135\times \mathit{\min}{\left(\frac{S_{cr}}{\kappa },1\right)}^{\alpha}\times \mathit{\max}{\left(\frac{S_{cr}}{\kappa },1\right)}^{-1.209}\\\times \mathit{\min}{\left(\frac{S_{cys}}{0.8},1\right)}^{-0.323}\times \mathit{\max}{\left(\frac{S_{cys}}{0.8},1\right)}^{-0.778}\\\times{0.9961}^{age}\times 0.963\left[ if\ female\right] \end{align*}

Where ${S}_{cr}$ is serum creatinine level in mg/dL; $\kappa$ is 0.7 for females and 0.9 for males; $\alpha$ is −0.219 for females and − 0.144 for males; ${S}_{cys}$ is serum cystatin C level in mg/L.

### GWAS for kidney biomarkers and function

We performed a GWAS on the 421 832 individuals with EUR genetic ancestry for creatinine levels, cystatin C levels, and eGFR measured using the four formulas described above on all biallelic variants with MAF > 1% (corresponding to 8.7 million variants). We performed 60 GWAS by combining these six different traits and ten different subsets of individuals. Specifically, we tested:

Six Traits:
  1. Serum Creatinine levels;
  2. Serum Cystatin C levels;
  3. The original creatinine-based CKD-EPI eGFR: eGFRcre (2009);
  4. The updated creatinine-based CKD-EPI eGFR: eGFRcre (2021);
  5. The cystatin C-based CKD-EPI eGFR: eGFRcys;
  6. The updated creatinine and cystatin C-based CKD-EPI eGFR: eGFRcrecys;
Ten groups of individuals divided according to their creatinine levels or CKD status
  1. Top 1%;
  2. Top 2%;
  3. Top 10%;
  4. Bottom 1%;
  5. Bottom 10%;
  6. Bottom 90%;
  7. Bottom 98%;
  8. Bottom 99%;
  9. Healthy individuals;
  10. Individuals diagnosed with CKD.

We used a linear regression model to perform the 60 GWAS. Specifically we utilized *plink* 2.3 [49] with parameters *—glm omit-ref log10 —no-parents —no-sex —no-pheno —maf 0.01 —hwe 0.000001 —max-alleles 2* to analyze only relatively common biallelic variants (MAF > 1%) in Hardy–Weinberg equilibrium (*P* > 1e-6). We used the top 10 genotype PCs, sex, and age as covariates.

The list of 836 genome-wide significant loci in the 60 GWAS was generated as follows:

1. For each GWAS, each genome-wide significant locus was expanded 1 Mb upstream and downstream of the lead variant;
2. We next used *bedtools merge -d 1 000 000* to merge all 2-Mb intervals that overlapped by at least one base pair.

To examine each pairwise combination of GWAS, we obtained all genome-wide significant loci for either tested trait, and the extracted all variants in these loci, regardless of their p-values. We then calculated the correlation of the effect sizes between all these variants.

### Annotating GWAS loci with putative gene targets

We obtained the coordinates of 62 492 genes from Gencode v34lift37 [50], corresponding to 763 881 unique coding sequences (CDSs), 1 381 709 exons, and 87 888 promoters. Using *bedtools closest -D ref* (51), we annotated each lead variant with its overlapping gene features (exon, intron or promoter) or, in case of intergenic variants, its closest gene.

### Overlap of the bottom99 dataset with GWAS catalog

We obtained the list of all the 487 214 associations between variants and traits or diseases included in the GWAS catalog (frozen at February 3, 2023) [26]. We selected only variants associated with creatinine levels, cystatin C levels and/or eGFR by filtering the ‘disease trait’ field for all entries that included ‘creatinine’, ‘cystatin C’ and ‘estimated glomerular filtration rate’, respectively.

To find correspondence between the GWAS lead variants in this study and the GWAS catalog for the creatinine, cystatin C and eGFRcrecys traits using the bottom99 dataset, we proceeded with multiple steps:

1. For each trait (creatinine, cystatin C and eGFRcrecys), we identified loci whose lead variant was included in the GWAS catalog and associated with the same trait;
2. For the remaining lead variants, we calculated LD with variants in the GWAS catalog using PLINK *—r2 square*. We annotated all variants in strong LD (r^2^ > 0.8) with variants in the GWAS catalog associated with the same trait.
3. For the remaining variants, we investigated whether their overlapping gene (in case of intergenic variants, the closest gene upstream and downstream of the tested variant) had been described in the GWAS catalog as associated with the same trait.
4. The remaining lead variants were considered as ‘novel’.

### Functional enrichment analysis of shared, creatinine-specific and cystatin C-specific GWAS loci

To perform functional enrichment analysis of the GWAS loci for the creatinine, cystatin C and eGFRcrecys traits using the bottom99 dataset, we annotated 465 loci with the gene closest to or overlapping the lead variant. We divided the genes into three sets: 1) shared: 216 genes overlapping loci shared between cystatin C and creatinine; 2) creatinine-specific: 137 genes overlapping creatinine loci but not cystatin C (46 genome-wide significant only for creatinine and 91 shared between creatinine and eGFRcrecys, but not cystatin C, Fig. 4D); and 3) 112 genes overlapping cystatin C-specific loci (48 genome-wide significant only for cystatin C and 64 shared between cystatin C and eGFRcrecys, but not creatinine, Fig. 4D).

To perform functional enrichment analysis, we used the enrichr [30] R package, which includes a comprehensive set of databases for: gene expression in relevant tissues and cell types (GTEx and Tabula Sapiens); gene function (Gene Ontology); pathways (WikiPathways and Reactome); and genetic associations (GWAS catalog). Specifically, we used the following ten enrichr datasets: GTEx_Tissues_V8_2023; GO_Biological_Process_2023; GO_Cellular_Component_2023; GO_Molecular_Function_2023; GWAS_Catalog_2023; WikiPathways_2019_Human; Reactome_2022; Tabula_Sapiens; GTEx_Tissue_Expression_Down; GTEx_Tissue_Expression_Up. For each gene set in each of the ten datasets, we tested the enrichment of our three gene sets using all human genes as background. P-values were calculated using the default enrichr test, which consists in combining the results from multiple tests [30].

## Supplementary Material

ckd_supplement_figures_V17_ddaf018

table_s1_ddaf018

table_s2_ddaf018

table_s3_ddaf018

table_s4_ddaf018

table_s5_ddaf018

table_s6_ddaf018

table_s7_ddaf018
