# Supplementary material for: Genetic analysis of elevated levels of creatinine and cystatin C biomarkers reveals novel genetic loci associated with kidney function
Source: Hum Mol Genet. 2025 Feb 10;34(9):751–64. doi: 10.1093/hmg/ddaf018 (PMC12010162; doi:10.1093/hmg/ddaf018)
Supplement: ckd_supplement_figures_V17_ddaf018 [file ckd_supplement_figures_v17_ddaf018.docx]

# Supplemental figures

## Figure S1: Correlation between different eGFR formulas


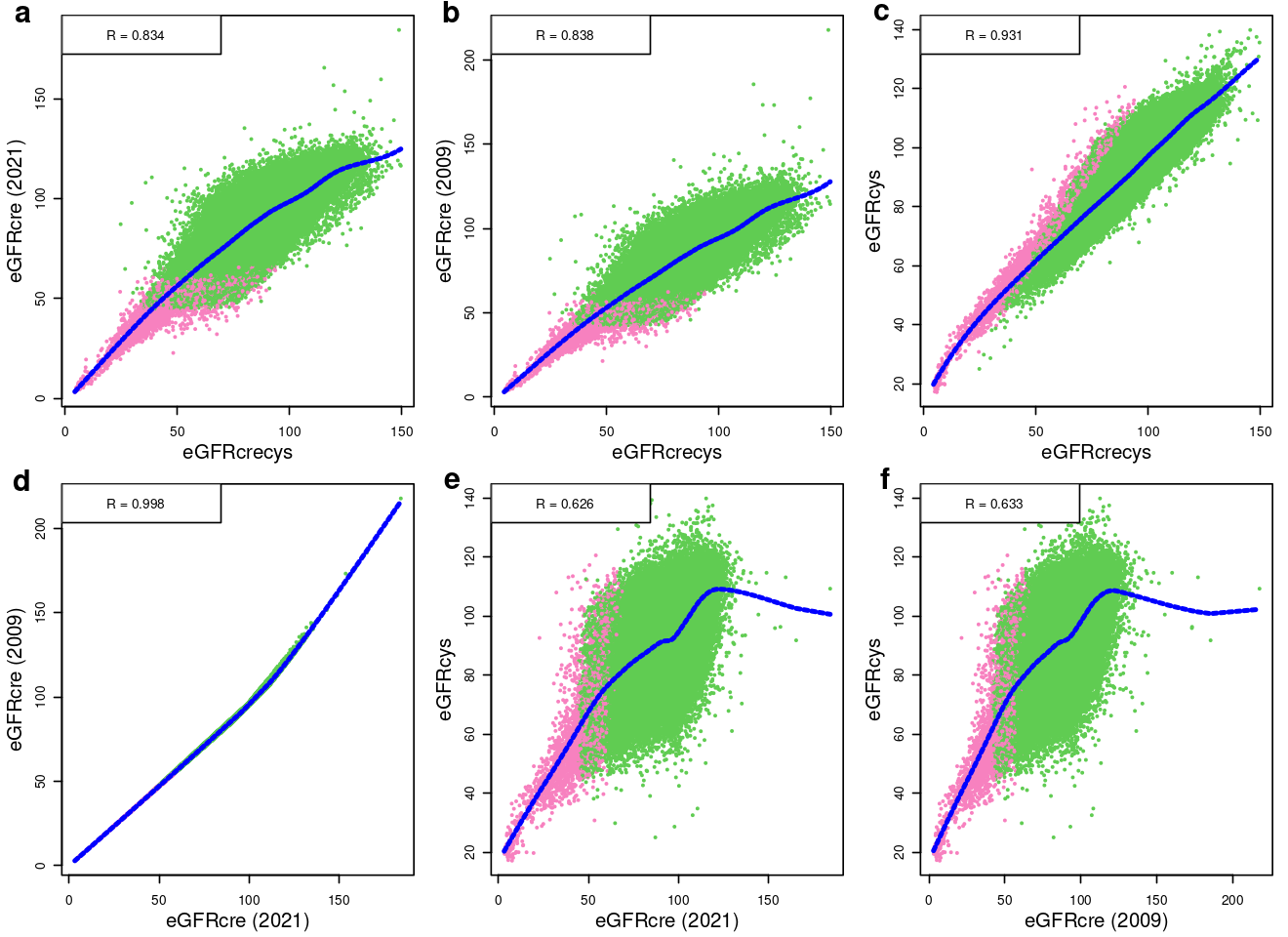


Scatter plots showing the correlation of eGFR levels calculated using the four different formulas. Overall, all measures of eGFR are strongly correlated, however, the two eGFR formulas that use only creatinine are the most strongly correlated (R = 0.998) than with the others. On the other hand, the eGFR measurements that only use creatinine are more weakly correlated with eGFRcys (R < 0.634). Individuals in the bottom99 (green) and top1 (pink) percentiles of creatine levels are indicated.

## Figure S2: QQ plots


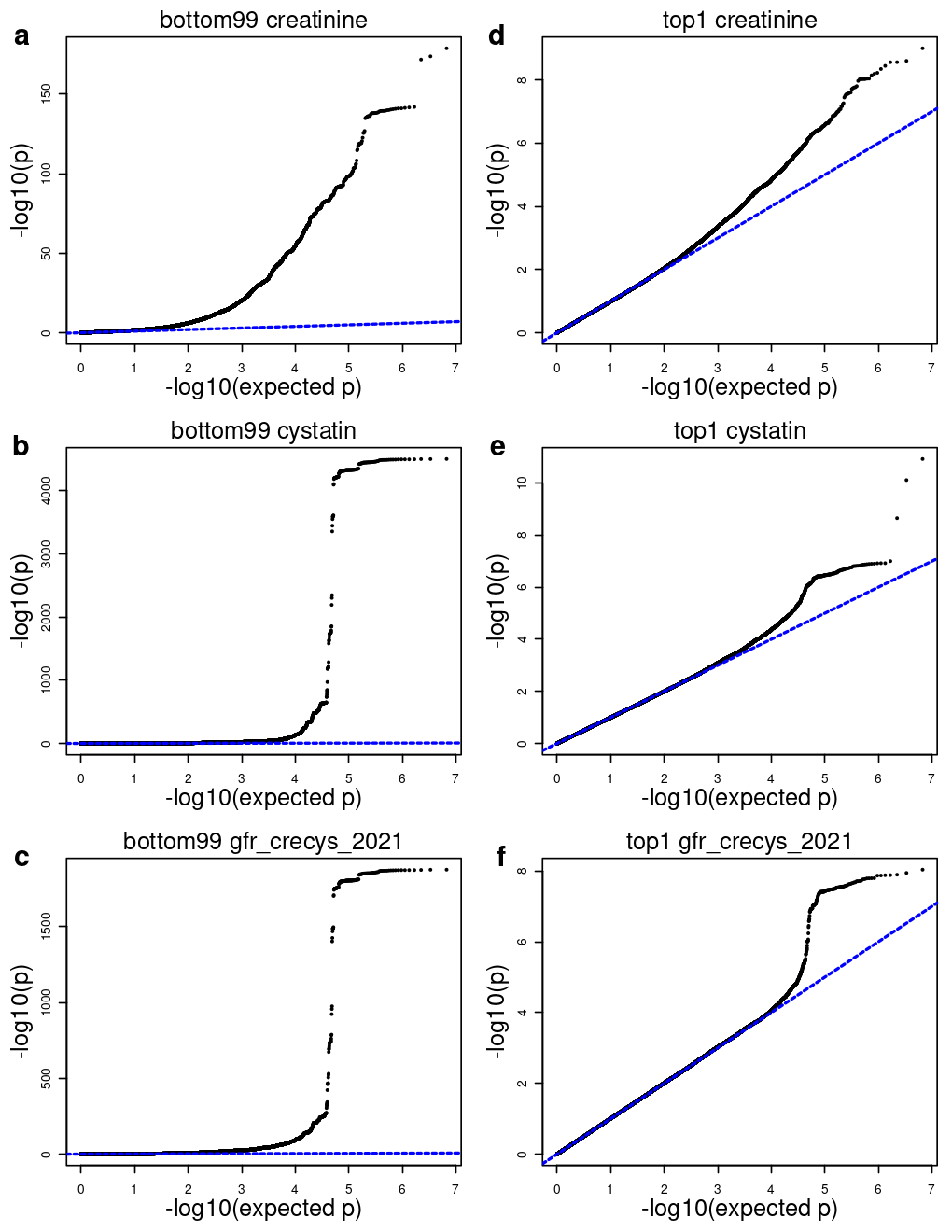


QQ plots of the GWAS p-values. As expected, based on the fact that the bottom99 dataset has 99 times more individuals compare with the top1 dataset, power for GWAS is very different, although the top1 dataset provides a strong deviation from the expected p-value distributions.

**Figure S3: GWAS of Creatinine, Cystatin C, and eGFRcrecys traits in the top1 set of individuals**


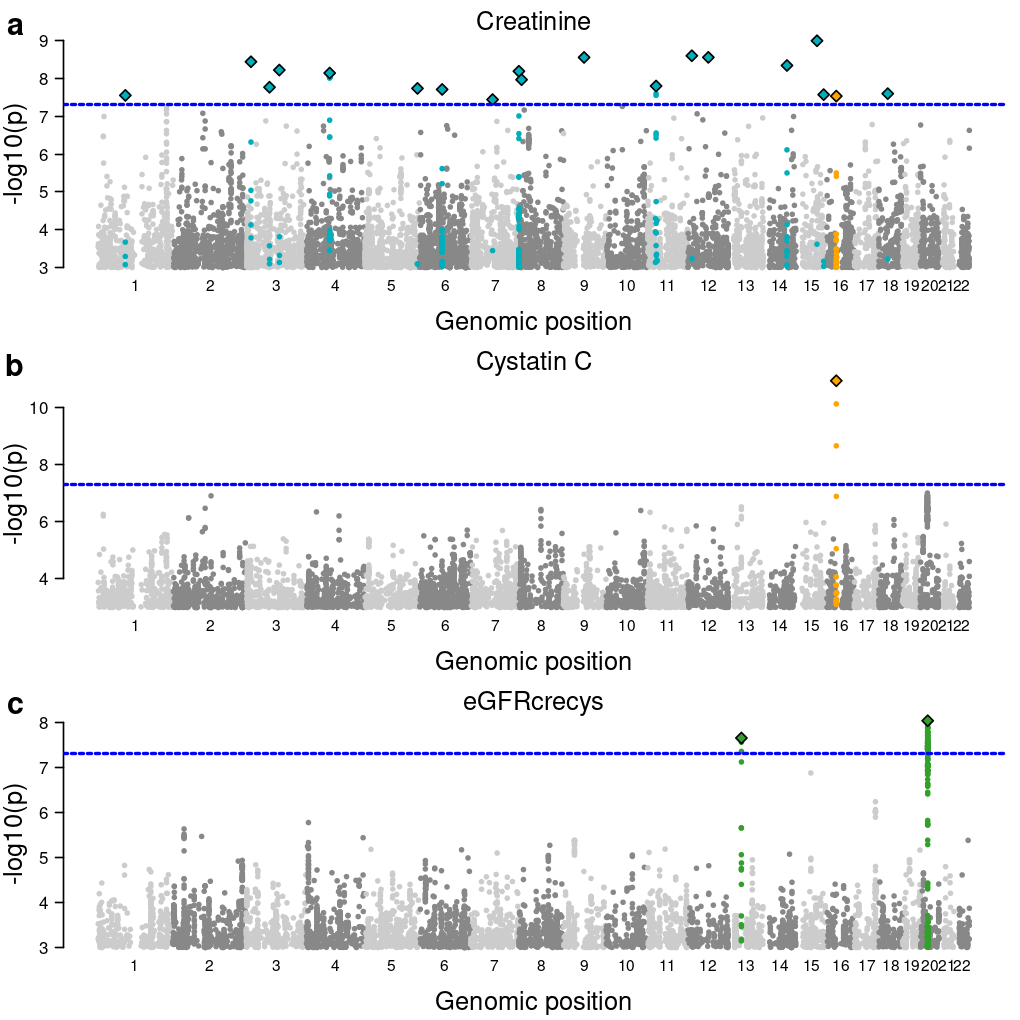


a-b) Manhattan plots showing the associations between genetic variation and a) creatinine, b) cystatin C levels and c) eGFRcrecys in the top1 set of individuals. Loci in orange are shared, and loci in blue are specific for creatinine and in green are specific for eGFRcrecys. Horizontal blue lines indicate significance (p < 1x10^-8^).

## Figure S4: Comparing self-reported with genetic ancestry


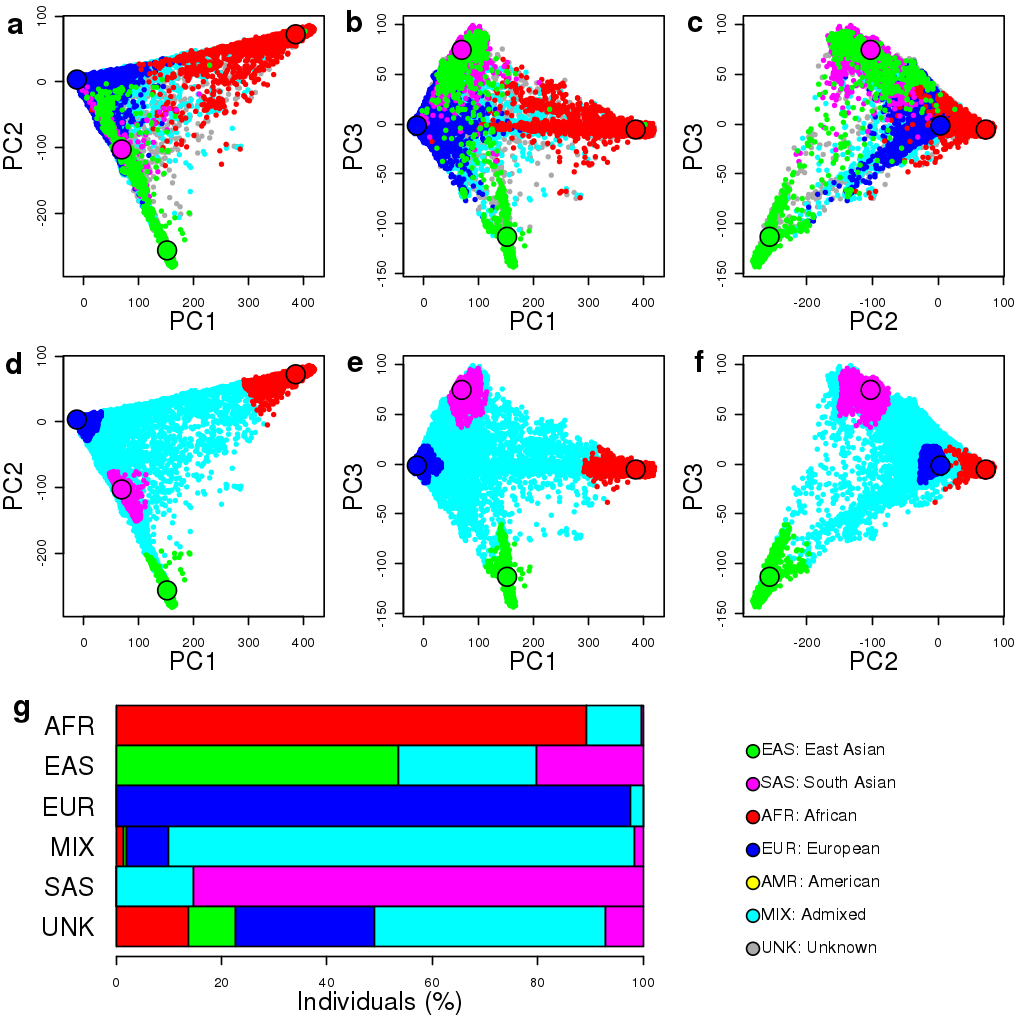


a-f) Genotype PCA plots for a, d) PC1 and PC2, b, e) PC1 and PC3 and c, f) PC2 and PC3. Individuals are colored according to a-c) self-reported ethnicity, and d-f) their global ancestry based on genotype PCA.

g) Stacked barplots showing, for each self-reported ethnicity, the distribution of genotype PCA-based global ancestry. As expected, individuals of unknown ethnicity and individuals who self-reported as admixed are more likely to be admixed than other individuals.

# Supplemental tables

## Table S1: associations between creatinine, cystatin C, and CKD incidence at each percentile

For each creatinine level percentile, shown are the correlation between creatinine and cystatin C levels and the percentage of individuals diagnosed with CKD.

## Table S2: correlation between creatinine levels and disease risk

The table shows the results from logistic regression between creatinine levels and chronic disease risk in the top1, bottom99 and bottom10 datasets. Shown are: the number of individuals and the number of patients diagnosed with each disease in each set, estimate, standard error and p-value calculated using the glm function in R. We further stratified the 4,017 individuals in the top1 dataset based on creatine levels and investigated if the positive relationship between higher creatinine levels and increased risk for all five chronic diseases was observable

## Table S3: Genome-wide significant loci

The table shows the genomic coordinates (chromosome, start, and end position) for each of the 836 genome-wide significant locus. Each subsequent column represents whether the locus had genome-wide significant variants in each of the 60 GWAS performed in this study (six traits: creatinine, cystatin C, eGFRcre (2009), eGFRcre (2021), eGFRcys and eGFRcrecys; ten datasets: top1, top2, top10, bottom1, bottom10, bottom90, bottom98, bottom99, healthy individuals and CKD patients).

## Table S4: Pairwise correlations between effect sizes at all GWAS loci

For each pairwise comparison between the 60 GWAS experiments, we show the correlation calculated on the effect sizes of all the variants at each genome-wide significant locus. For comparisons where neither GWAS had genome-wide significant loci, the third column (displaying the correlation between effect sizes) is empty.

## Table S5: Overlap with GWAS catalog

For each locus associated with creatinine, cystatin C and eGFR in the bottom99 dataset, shown are: coordinates of the locus and the lead variant, the reference and alternative alleles, rsID, minor allele frequency, the overlapping gene, the number (n) of individuals in each GWAS, effect size beta, its standard error and -log_10_ (p-value). We also show whether the lead variant was **novel** (not associated with creatinine levels, cystatin C or eGFR levels in the GWAS catalog) or in the GWAS catalog: 1) lead variant **in catalog**; 2) lead variant **in LD** with with GWAS catalog variant (the rsID of the variant in the GWAS catalog is indicated in column R and its R^2^ in column S); or 3) the overlapping or nearest gene (**same gene**) to the lead variant was in the GWAS catalog in column T: True, False.

## Table S6: Functional enrichment analysis of GWAS loci shared between creatinine and cystatin C, creatinine-specific and cystatin C-specific

We performed functional enrichment analysis on three gene sets: 1) 216 genes overlapping loci shared between cystatin C and either creatinine and/or eGFRcre; 2) 137 genes overlapping creatinine loci but not cystatin C; and 3) 112 genes overlapping cystatin C-specific loci. For each set of loci (creatinine-specific, cystatin C-specific or shared), the table describes: the functional database (GTEx_Tissues_V8_2023; GO_Biological_Process_2023; GO_Cellular_Component_2023; GO_Molecular_Function_2023; GWAS_Catalog_2023; WikiPathways_2019_Human; Reactome_2022; Tabula_Sapiens; GTEx_Tissue_Expression_Down; GTEx_Tissue_Expression_Up), the gene set, p-value, Benjamini-Hochberg-adjusted p-value and odds ratio compared with background (all human genes).

## Table S7: GWAS lead variants in the top1 dataset

The table shows the summary statistics for the 22 GWAS lead variants across all three traits (creatinine, cystatin C and eGFRcrecys) in the top1 dataset. Shown are the genomic coordinates, rsID, reference and alternative alleles, effect size, standard error and -log10(p-value).
